# Alphaviruses in Cancer Therapy

**DOI:** 10.3389/fmolb.2022.864781

**Published:** 2022-04-14

**Authors:** Kenneth Lundstrom

**Affiliations:** Pan Therapeutics, Lutry, Switzerland

**Keywords:** self-replicating RNA, recombinant particles, RNA replicon, DNA replicon, cancer vaccine, immunotherapy

## Abstract

Alphaviruses have been engineered as expression vectors for different strategies of cancer therapy including immunotherapy and cancer vaccine development. Administration of recombinant virus particles, RNA replicons and plasmid DNA-based replicons provide great flexibility for alphavirus applications. Immunization and delivery studies have demonstrated therapeutic efficacy in the form of reduced tumor growth, tumor regression and eradication of established tumors in different animal models for cancers such as brain, breast, colon, cervical, lung, ovarian, pancreas, prostate cancers, and melanoma. Furthermore, vaccinated animals have showed protection against challenges with tumor cells. A limited number of clinical trials in the area of brain, breast, cervical, colon prostate cancers and melanoma vaccines has been conducted. Particularly, immunization of cervical cancer patients elicited immune responses and therapeutic activity in all patients included in a phase I clinical trial. Moreover, stable disease and partial responses were observed in breast cancer patients and prolonged survival was achieved in colon cancer patients.

## Introduction

Although significant progress has been made on many fronts of cancer treatment, the continuous increase in cancer cases because of pollution, unhealthy eating and lifestyle choices, and an aging population keep the suffering and mortality rates high (Magee et al., 2013). In addition to conventional chemo- and radiotherapy approaches, non-viral and viral based cancer therapies have been applied (Lundstrom and Boulikas, 2003). One approach has been to deliver cytotoxic or suicide genes (Zarogoulides et al., 2013) or anti-tumor genes (Liu et al., 2005) with the aim of killing tumor cells while normal tissue is unaffected. Several viruses have been referred as oncolytic demonstrating natural tumor targeting and specific replication in tumor cells leading to their death without affecting normal cells. For example, the M1 alphavirus possesses natural oncolytic activity (Zhang et al., 2021) and Sindbis virus (SIN) has showed natural tumor targeting (Tseng et al., 2004). Cancer immunotherapy has caught plenty of attention recently although its first use dates to William B. Coley’s discovery of tumor regression in inoperable bone sarcoma after bacterial injection (Coley 1891; Kozlowska et al., 2013). The modern approach of cancer immunotherapy aims at boosting or restoring the ability of the immune system to detect and destroy cancer cells (Scott et al., 2012). Cancer immunotherapy comprises administration of various cytokines, and expression of tumor-associated antigens (TAAs) (Fortner et al., 2017). The advantage of using viral vectors relates to their features of excellent delivery and high level of recombinant protein expression. On the other hand, viral vectors can pose a safety risk, which has triggered the engineering of replication-deficient and suicide vectors. A variety of viral vectors based on adenoviruses, adeno-associated virus (AAV), alphaviruses, herpes simplex virus (HSV), lentiviruses (LV), measles virus (MV), Newcastle disease virus (NDV), and rhabdoviruses have demonstrated promising results in both preclinical animal models and clinical trials (Lundstrom, 2022). An essential part of viral vector-based cancer therapy has relied on the application of oncolytic viruses, which can provide selective killing of tumor cells while causing only minor or no damage to normal tissue (Kaufman et al., 2015). In this review, the focus will be on alphaviruses and their application in cancer therapy.

## Alphavirus Vectors

**FIGURE 1:**
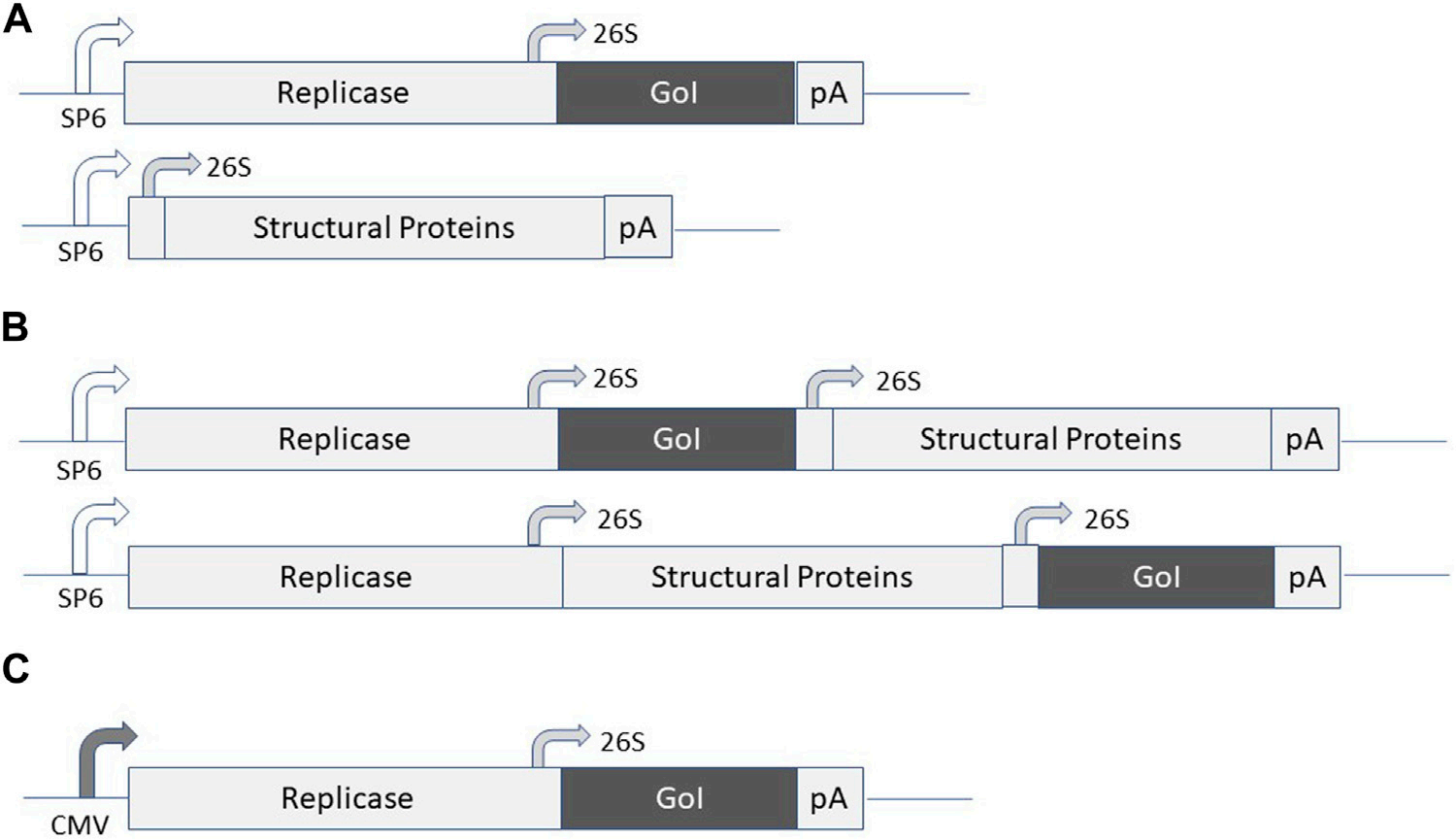
Expression systems for alphaviruses. **(A)** Replication-deficient alphavirus particles. The alphavirus expression vector contains the non-structural protein (nsP) genes, the subgenomic 26S promoter, the gene of interest (GoI) and the poly A signal. The helper vector contains the subgenomic promoter, the structural protein (C-p62-6K-E1) genes and the poly A signal. SP6 RNA polymerase is used for *in vitro* transcription of RNA from expression vector and helper vector DNA and co-transfected/electroporated into BHK-21 cells for virus production. **(B)** Replication-proficient alphavirus particles. SP6 RNA polymerase is used for *in vitro* transcription of full-length alphavirus RNA genome including the GoI introduced either upstream or downstream of the structural protein genes followed by transfection/electroporation into BHK-21 cells for virus production. **(C)** DNA/RNA layered vector. The plasmid DNA replicon is transfected into mammalian cells for expression of the GoI.

Alphavirus vectors have been engineered for the expression of recombinant proteins in mammalian cells lines, gene therapy applications and vaccine development (Lundstrom 2015). Briefly, alphaviruses are enveloped single-stranded RNA viruses (Strauss and Strauss, 1994). Due to the positive polarity of the genome, the ssRNA is directly translated in the cytoplasm of infected host cells. Expression of the alphavirus non-structural genes generates the replicase complex resulting in self-replication of RNA, production of structural proteins, encapsulation of RNA genomes, and assembly and release of new viral particles. The most commonly used expression vector systems are based on Semliki Forest virus (SFV) (Liljestrom and Garoff, 1991), SIN (Xiong et al., 1989) and Venezuelan equine encephalitis virus (VEE) (Davis et al., 1989). In principle, alphavirus vectors can be delivered as recombinant virus particles, RNA replicons or layered DNA/RNA plasmid vectors (Figure 1). In the case of viral particle delivery both replication-deficient and -proficient particles have been engineered. In the former case, the alphavirus genome is split on two or more plasmid vectors, where the expression vector carries the alphavirus non-structural genes (nsP1-4) and the gene of interest (GoI) and the structural genes are placed on one or several (split helper) helper plasmids. Recombinant virus particles are generated by *in vitro* transcription of RNA from linearized DNA plasmids followed by electroporation or transfection of mammalian cells such as BHK-21 cells (Figure 1A). Replication-proficient alphavirus particles are produced by introduction of the GoI in the full-length alphavirus genome downstream of either the nsP genes or the structural genes (Figure 1B). Alternatively, alphavirus vectors can be delivered as naked RNA, but due to the sensitivity of single-stranded RNA (ssRNA) to degradation, the delivery and stability of RNA can be significantly improved by encapsulation in lipid nanoparticles (Geall et al., 2012; Blakney et al., 2019). Moreover, DNA replicon vectors have been engineered by replacing the SP6 RNA polymerase promoter with a CMV promoter (DiCiommo and Bremner, 1998) (Figure 1C). In all cases, the vectors possess the self-replicating feature of single-stranded RNA viruses due to the presence of the nsP-based replicon complex, which can accumulate approximately 10^6^ copies of viral RNA per cell in the cytoplasm of infected cells (Frolov et al., 1996). The RNA amplification in combination with the strong 26S subgenomic promoter present in alphavirus vectors generate high levels of GoI such as TAA or cytotoxic gene expression. Because of the degradation of the alphavirus ssRNA, only transient expression and no integration into host cell genome occur, ideal features for vaccine development. Concerning DNA replicons, the potential chromosomal integration risk is as low as determined for conventional DNA plasmids (Langer et al., 2013).

## Alphavirus Vectors in Tissue-Specific Cancer Therapy

Alphavirus vectors have been frequently used for cancer therapy in various animal models and to some extent in clinical trials. A common approach has been to overexpress TAAs to provide both therapeutic and prophylactic effects. In many cases, green fluorescent protein (GFP) and luciferase have been used as reporter genes to monitor location and delivery efficacy. Therapeutic activity has been achieved through alphavirus-based expression of cytotoxic and anti-tumor genes, but also due to the alphavirus induced apoptosis (Olkkonen et al., 1993). However, apoptotic events are not restricted to tumor cells as they also occur in normal cells infected by alphaviruses, which suggests that for systemic alphavirus administration it is recommended to use oncolytic or tumor-targeted viruses. Moreover, immunostimulatory and particularly cytokines have been targeted for cancer immunotherapy. Another approach comprises the application of oncolytic viruses for the therapy of existing tumors (Fukuhara et al., 2016). Examples of the above-mentioned strategies are described below and summarized in Table 1.

**TABLE 1:** Examples of Alphavirus applications in cancer therapy.

Cancer	Vector	Strategy	Response	Ref
Brain				
GBM	SFV-Endostatin	VLPs	Tumor regression in mice	Yamanaka et al. (2001)
GBM	SFV-IL-18	VLPs + IL-12	Anti-tumor and protective immunity in mice	Yamanaka et al. (2003)
RG2	SFV-IL-12	VLPs	70–87% reduction in tumor volume in mice	Roche et al. (2010)
GBM	SIN-gp100/IL-18	DNA	Enhanced protection, prolonged survival	Yamanaka and Xanthopoulos, (2005)
GBM	SFV VA-GFP	Oncolytic SFV	Single injection > long-term survival	Heikkilä et al. (2010)
CT-2A	SFV4-miRT124	RPVPs	Tumor inhibition, prolonged survival	Martikainen et al. (2015)
GBM	LSFV-IL-12	LSFV	Phase I/II protocol for recurrent GBM	Ren et al. (2003)
Breast				
A2L2	SIN-HER2/neu	DNA + Ad-neu	Prolonged survival in mice	Wang et al. (2005b)
A2L2	SIN-HER2/neu	DNA	Tumor protection with 80% less DNA	Lachman et al. (2001)
A2L2	VEE-HER2 ECD/TM	VRPs	Complete prevention of tumor formation	Wang et al. (2005a)
HER2	VEE-HER2 ECD/TM	VRPs	Phase I: PR: 1 patient, SD; 2 patients	NCT03632941
HER2	VEE-HER2 ECD/TM	VRPs + Pembro	Phase II: study in progress	Crosby et al. (2019)
4T1	SFV-IL-12	VLPs + LVR01	Superior inhibition of metastases	Kramer et al. (2015)
TNBC	M1	oM1 + Dox	Enhanced anti-tumor activity with Dox	Zhang et al. (2021)
Cervical				
HPV16	VEE.HPV16 E7	VLPs	Protection against tumor challenges	Velders et al. (2001)
HPV16	SIN AR339	Oncolytic SIN	Significant tumor regression	Unno et al. (2005)
HPV16	SFVenh-HPV-E6-E7	VLPs	Complete eradication of tumors in mice	Daemen et al. (2003)
HPV16	SFV-sHELP-E7SH	VLPs	Tumor regression, protection of mice	Ip et al. (2015)
HPV16	SFV-HPV-E-E7	DNA	85% of immunized mice tumor free	Van der Wall et al. (2018)
HPV16	SFV HPV-E6-E7	VLPs (Vvax001)	Phase I: immune response in all patients	Komdeur et al. (2021)
Colon				
MC38	SFVenh-IL-12	VLPs	Complete tumor regression in 80% of mice	Rodriguez-Madoz et al. (2005)
MC38	SFV-IL-12	VLPs + anti-PD1	Synergism with immune checkpoint blockade	Quetglas et al. (2015)
MC38	VEE-IL-12/CEA	VLPs	Superior combination therapy in mice	Lyons et al. (2007)
CT26	SFV-VEGFR-2/IL-4	VLPs	Prolonged survival after combination	Ying et al. (1999)
CT26	SFV-LacZ	RNA	Immunogenicity, prolonged survival	Crosby et al. (2020)
CC	VEE-CEA	VLPs	Phase I: Prolonged overall survival	Osada et al. (2012)
Lung				
H358a	SFV-EGFP	VLPs	Complete regression of tumors	Murphy et al. (2000)
A549	SFV VA-EGFP	Oncolytic SFV	Superiority to adenovirus in mice	Määttä et al. (2008)
CL25	SIN-LacZ	VLPs	Complete remission, prolonged survival	Granot et al. (2014)
Melanoma				
B16	VEE-TRP-2	VLPs	Immune response, prolonged survival	Avogadri et al. (2010)
B16	VEE-TRP-2	VLPs + CTLA-4	Tumor regression in 50% of mice	Avogadri et al. (2014)
B16	VEE-TRP-2	VLPs + GITR	Tumor regression in 90% of mice	Avogadri et al. (2014)
B16	SFV-VEGFR-2/IL-12	Combination of 2	Combination of SFV-VEGFR-2/IL-12 +	Yin et al. (2015)
	SFV-surv/βhCG	DNA vectors	SFV-surv/βhCG superior	
B16-OVA	SFV-IL-12	VLPs + anti-PD1	Synergism with immune checkpoint blockade	Quetglas et al. (2015)
MIII-IV	LSFV-IL-12	LSFV	Phase I: safe, 10-fold increase in IL-12	Ren et al. (2003)
Ovarian				
C33A	SIN AR339	Oncolytic SIN	Suppressed ascites formation in mice	Unno et al. (2005)
ES2	SIN-IL-12	VLPs + irinotecan	Long-term survival in mice	Granot & Meruelo (2012)
MOSEC	SFV-OVA	VLPs + VV	Enhanced anti-tumor activity	Zhang et al. (2010)
Pancreatic				
MePC	VEE-CEA	VLPs	Prolonged survival in patients	Morse et al. (2010)
LAPC	M1	oM1 + IRE	Prolonged survival in mice	Sun et al. (2021)
Prostate				
TRAMP	VEE-PSMA	VLPs	Th1-biased response, CTL activity	Durso et al. (2007)
CRPC	VEE-PSMA	VLPs	Phase I: safe, weak immunogenicity	Slovin et al. (2013)
TRAMP-C	VEE-STEAP	conDNA + VLPs	Prolonged survival, protection in mice	Garcia-Hernandez et al. (2007)
TRAMP	VEE-PSCA	VLPs	Long-term survival in 90% of mice	Garcia-Hernandez et al. (2008)

A2L2, breast cell line expressing HER2; Ad-neu, Adenovirus-neu; anti-PD1, anti-PD1 monoclonal antibody; CEA, carcinoembryonic antigen; CC, colon cancer; conDNA, conventional plasmid DNA; CRPC, castration resistant prostate cancer; CTLA-4, CTL antigen-4; Dox, doxorubicin; GBM, glioblastoma multiforme; GITR, glucocorticoid-induced tumor necrosis factor receptor; HPV, human papillomavirus; IRE, irreversible electroporation; LAPC, locally advanced pancreatic cancer; LSFV, liposome encapsulated SFV particles; LVR01, *Salmonella typhimurium* aroC strain; M1, oncolytic M1 alphavirus; MePC, metastatic pancreatic cancer; MOSEC, murine ovarian surface epithelial carcinoma; OVA, ovalbumin; Pembro, Pembrolizumab; PR, partial response; PSCA, prostate stem cell antigen; PSMA, prostate specific membrane antigen; RG2, rat glioma 2; RPVPs, replication-proficient viral particles; SD, stable disease; SFV, Semliki Forest virus; SIN, Sindbis virus; TNBC, triple-negative breast cancer; TRP-2, tyrosine-related protein-2; STEAP, six-transmembrane epithelial antigen of the prostate; TRAMP, transgenic adenocarcinoma of the prostate; VEE, Venezuelan equine encephalitis virus; VEGFR-2, vascular endothelial growth factor-2; VLPs, virus-like particles; VV, vaccinia virus.

Among the different cancer types, brain tumors, especially glioblastomas have been targeted for alphavirus-based therapy. Recombinant SFV particles expressing endostatin (SFV-Endostatin) was compared to retrovirus-endostatin and SFV-LacZ particles based on their oncolytic activity in a B16 mouse brain tumor model (Yamanaka et al., 2001). The SFV-Endostatin particles provided superior tumor growth inhibition and reduced intratumoral vascularization compared to the other treatments. Moreover, endostatin serum levels were 3-fold higher after intravenous administration of SFV-Endostatin particles compared to intravenous endostatin. SFV particles expressing interleukin-18 (IL-18) were also applied for transduction of dendritic cells (DCs) combined with systemic administration of IL-12 (Yamanaka et al., 2003). The combination therapy was superior to IL-12 alone generating enhanced Th1 responses in tumor-specific CD4^+^ and CD8^+^ T cells and natural killer cells in mice with B16 brain tumors. Moreover, the anti-tumor and protective immunity were stronger. SFV particles expressing IL-12 have been evaluated in a syngeneic RG2 rat glioma model (Roche et al., 2010). Administration of a low dose of 5 × 10^7^ SFV-IL-12 particles via an implanted cannula resulted in a 70% reduction in tumor volume and a significant prolongation of survival. The high dose of 5 × 10^8^ SFV-IL-12 particles generated an 87% reduction in tumor volume but could also potentially induce vector-related lethal pathology. In another approach, SIN DNA replicons expressing the human gp100 and mouse IL-18 were intramuscularly administered to mice bearing B16-gp100 brain tumors (Yamanaka and Xanthopoulos, 2005). Co-delivery of SIN-gp100 and SIN-IL-18 DNA replicons enhanced the therapeutic and protective effect against brain tumors and significantly prolonged the survival of mice. The replication-proficient SFV(A774nsP) vector expressing enhanced green fluorescent protein (VA-EGFP) has demonstrated oncolytic properties in a subcutaneous orthotopic tumor model in BALB/c mice (Heikkilä et al., 2010). Stable expression of firefly luciferase (Luc) was completely inhibited in mice receiving a single intravenous injection of SFV VA-EGFP. Furthermore, 16 out of 17 immunized animals showed long-term survival. Therapeutic gene therapy applications of alphaviruses, particularly replication-proficient vectors, for brain tumors have presented some concerns because of their neurovirulence (Griffin 2016). In this context, the distribution of SFV particles and naked RNA replicons expressing Luc was compared in tumor-free and 4T1 mammary tumor-bearing mice after intravenous, intraperitoneal or intratumoral administration (Vasilevska et al., 2012). Intravenous administration of SFV-Luc RNA showed primary brain targeting in both mice without tumors and 4T1 tumor-bearing mice. In contrast, intratumoral injection led to high Luc expression in tumors. Intravenous and intraperitoneal administration of a high dose of SFV-Luc particles (6 × 10^9^ particles/ml) demonstrated a broad distribution of expression, whereas a reduced viral dose (2 × 10^8^ particles/ml) resulted in predominant tumor targeting. Furthermore, the neurotrophic affinity of SFV particles has been addressed by insertion of neuron-specific micro-RNA miRT124 sequences into the replication-proficient SFV4 vector (Martikainen et al., 2015). Significant tumor growth inhibition and prolonged survival was seen in C57BL/6 mice with CT-2A orthotopic gliomas after a single intraperitoneal injection of SFV4-miRT124 particles. No clinical studies on alphavirus-based treatment of brain tumors have been published, so far. However, a phase I/II protocol for the treatment of patients with recurrent glioblastoma with liposome encapsulated SFV particles expressing IL-12 (LSFV-IL-12) has been published (Ren et al., 2003). According to the protocol, the plan is to administer by continuous intratumoral infusion doses of 1 × 10^7^ to 1 × 10^9^ infectious particles.

Breast cancer is another indication, which has received attention as a therapeutic target. In one study, the HER2/neu gene was expressed from a SIN DNA replicon and an adenovirus vector (Wang et al., 2005b). SIN-HER2/neu DNA and Adeno-neu particles were administered into the mammary fat pad of mice or intravenously as a model for lung metastases. Immunization with SIN-HER2/neu DNA or Adeno-neu particles prior to tumor challenges with A2L2 cells expressing the rat HER2/neu gene showed a significant inhibition of tumor growth. In contrast, immunization with either vector 2 days after the tumor challenge was inefficient. However, a prime immunization with SIN-HER2/neu DNA followed by an Adeno-neu particle booster led to a significant prolongation of the survival of mice. Moreover, in comparison to a conventional DNA plasmid, intradermal administration of SIN-HER2/neu DNA replicons required 80% less DNA to elicit robust antibody responses and protection against tumor challenges in BALB/c mice (Lachman et al., 2001). VEE virus-like replicon particles (VRPs) expressing the extracellular domain (ECD) and transmembrane (TM) domains of HER2 were evaluated in a transgenic HER2 mouse model (Wang et al., 2005a). The VEE-HER2 VRPs prevented or inhibited the growth of HER2/neu-expressing mouse breast cancer cells either after injection into mammary tissue or administered intravenously. High levels of neu-specific CD8^+^ T lymphocytes and serum IgG were obtained. Moreover, complete prevention of tumor formation was seen in immunized mice. VEE-HER2 VRPs have been subjected to a phase I clinical trial in stage IV HER2 overexpressing breast cancer patients (NCT03632941). The immunization showed good tolerance and resulted in partial response (PR) in one patient and stable disease (SD) in two other patients. Additionally, a combination therapy phase II trial with VEE-HER2 VRPs and pembrolizumab combination in HER2-positive breast cancer patients has started (Crosby et al., 2019). In another approach, mice with 4T1 mammary tumor nodules were immunized with 2 × 10^8^ SFV-IL-12 particles and 2 × 10^7^ units of the *Salmonella typhimurium* aroC strain (LVR01) (Kramer et al., 2015). The treatment resulted in complete inhibition of lethal lung metastasis formation and provided long-term survival in 90% of vaccinated mice. The synergistic effect of SFV-IL-12 and LVR01 was superior to immunization with either agent alone. The oncolytic M1 alphavirus has been applied for experimental treatment of triple-negative breast cancer (TNBC), the most aggressive molecular subtype of breast cancer (Zhang et al., 2021). The oncolytic effect of M1 could be enhanced by 100-fold by co-administration of doxorubicin *in vitro* and it also reduced significantly tumor growth *in vivo*.

In the context of prophylactics and therapeutics against cervical cancer, the human papilloma virus (HPV) recombinant protein-based vaccine Gardasil was approved in 2006 by the FDA (http://www.fda.gov/NewsEvents/Newsroom/PressAnnouncements/2006/ucm108666.htm). The viral vector-based vaccine platform has, however, been pursued because of the rapid high-level short-term transgene expression, favorable for vaccine development. For instance, VEE particles expressing the HPV16 E7 protein were administered to C57BL/6 mice, which induced robust CD8^+^ T cell responses and provided protection against tumor challenges (Velders et al., 2001). Moreover, the replication-proficient SIN AR339 strain induced cytopathogenicity and apoptosis in HeLaS3 and C33A cancer cells, but not in normal keratinocytes *in vitro* (Unno et al., 2005). A single intraperitoneal or intravenous injection of SIN AR339 generated significant regression of established cervical tumors in nude mice. In the context of SFV, the HPV E6-E7 fusion was introduced into the SFVenh vector containing the translation enhancer signal from the SFV capsid gene to improve expression levels and immunogenicity (Daemen et al., 2003). Immunization of C57BL/6 mice showed a complete eradication of established tumors and a long-term high-level CTL activity lasting up to 340 days. In another approach to enhance immunogenicity, helper T-cell epitopes and an endoplasmic reticulum (ER) targeting signal were fused to the HPV E6 and E7 proteins (Ip et al., 2015). Immunization of C57BL/6JOlaHsd mice with as few SFV-sHELP-E7SH particles as 1 × 10^5^ resulted in tumor regression and protection against challenges with tumors. SFV DNA replicons expressing HPV E6-E7 have also been applied for intradermal immunization of C57BL/6 mice followed by electroporation (van de Wall et al., 2018). In comparison to immunization with a conventional DNA vector, which did not prevent tumor growth, a 200-fold lower dose (0.05 µg) of the SFV HPV E6-E7 DNA replicon rendered 85% of mice tumor free. In the context of clinical trials, in a phase I study 12 individuals with a history of cervical intraepithelial neoplasia were immunized with doses of 5 × 10^5^, 5 × 10^6^, 5 × 10^7^, or 2.5 × 10^8^ of the SFVenh-HPV E6/E7 vaccine candidate (Vvax001) (Komdeur et al., 2021). The immunization was determined safe resulting in HPV-specific immune responses in all 12 patients.

Alphavirus vectors have also been used for colon cancer therapy. For example, IL-12 has been expressed from SFV vectors for evaluation in a poorly immunogenic MC38 colon adenocarcinoma mouse model (Rodriguez-Madoz et al., 2005). The two subunits of IL-12 were either expressed from a single subgenomic promoter from the SFV-IL-12 vector or from two independent 26S promoters from the SFVenh-IL-12 vector. In the latter case, IL-12 was expressed at 8-fold higher levels. A single intratumoral administration of 1 × 10^8^ particles of either SFV-IL-12 or SFVenh-IL-12 resulted in complete tumor regression and long-term tumor-free survival in mice with implanted MC38 xenografts. The SFVenh-IL-12 induced more efficiently anti-tumor responses at lower doses compared to SFV-IL-12. The anti-tumor response was increased after repeated intratumoral injections and was superior to first generation adenovirus vectors in elimination of tumors. Furthermore, co-administration of SFV-IL-12 particles and the anti-PD1 monoclonal antibody showed a synergistic effect in the MC38 mouse tumor model (Quetglas et al., 2015). Similarly, the superiority of the combination therapy was demonstrated in a B16-OVA melanoma mouse model (Quetglas et al., 2015). In the case of VEE, C57BL/6 mice with MC38-CEA-2 tumors were immunized with VEE-IL-12 particles and VEE particles expressing the carcinoembryonic antigen (CEA) (Osada et al., 2012). VEE-IL-12 immunization induced stronger immune responses than administration of IL-12 protein. The anti-tumor activity and survival time were superior after immunization with both VEE-IL-12 and VEE-CEA compared to VEE-IL-12 or VEE-CEA alone. SFV particles carrying the vascular endothelial growth factor receptor-2 (VEGFR-2) were evaluated in a CT26 colon carcinoma mouse model (Lyons et al., 2007). A significant inhibition of tumor growth and metastatic spread was seen in mice either prophylactically or therapeutically immunized with SFV-VEGFR-2 particles. However, co-immunization with SFV-IL-12 particles completely abrogated antibody responses and anti-tumor activity seen for SFV-VEGFR-2 particles alone. In contrast, co-administration of SFV-VEGFR-2 and SFV-IL-4 particles enhanced VEGFR-2-specific antibody titers and extended survival of mice after tumor challenges compared to immunization with only SFV-VEGFR-2 particles. SFV RNA replicons have also been evaluated. In this context, a single intramuscular immunization of mice with 0.1 µg of SFV-LacZ RNA replicons elicited robust antigen-specific immune responses and protected mice from tumor challenges due to apoptotic activity (Ying et al., 1999). Moreover, the survival of mice with pre-existing tumors was extended after immunization with SFV-LacZ RNA replicons. In the case of clinical trials, a phase I study was conducted in patients with stages III and IV colorectal cancer immunized with VEE-CEA particles four times every 3 weeks (Crosby et al., 2020). The treatment induced antigen-specific immune responses and long-term survivors were identified among both stage III and IV patients indicating an extended overall survival.

In the case of lung cancer, SFV-EGFP particles demonstrated efficient killing of human H358a non-small cell lung cancer (NSCLC) cells and inhibition of H358a spheroid growth (Murphy et al., 2000). SFV-EGFP particles were injected into nu/nu mice with H358a xenografts resulting in complete tumor regression in three out of seven immunized mice. Replication-proficient SFV VA-EGFP particles were compared to the conditionally replicating adenovirus Ad-Delta24TK-GFP vector in nude mice with A549 lung adenocarcinoma implants (Määttä et al., 2008). The study demonstrated superior survival of mice locally immunized with SFV VA-EGFP. Neither vector system did elicit significant immune responses after systemic administration. Also, SIN particles have been subjected to studies in lung cancer models. Intravenous administration of SIN-LacZ particles to CT26.CL25 colon tumor-bearing mice showed complete tumor remission and provided long-term survival (Granot et al., 2014).

Melanoma is a cancer indication that has received plenty of attention, including prophylactic and therapeutic applications of alphaviruses (Zappasodi and Merghoub, 2015). In this context, VEE particles expressing the tyrosine-related protein-2 (TRP-2) showed humoral immune responses, anti-tumor activity and prolonged survival in a B16 mouse melanoma model (Avogadri et al., 2010). Combination therapy of VEE-TRP-2 with antagonist anti-CTL antigen-4 (CTLA-4) or agonist anti-glucocorticoid-induced tumor necrosis factor receptor (GITR) monoclonal antibodies (mAbs) led to tumor regression of 50 and 90% of mice, respectively (Avogadri et al., 2014). Additionally, combination therapy with two SFV DNA replicons expressing VEGFR-2 and IL-12 from one DNA replicon and survivin and β-hCG antigens from another DNA replicon showed tumor growth inhibition and prolonged survival in mice with B16 melanoma xenografts (Yin et al., 2015). The combination therapy showed superiority to immunization with either SFV DNA replicon alone. In the context of clinical evaluation, 18 patients with stage III or IV metastasizing melanoma and renal cell carcinoma have been subjected to intravenous administration of 1 × 10^8^ or 1 × 10^9^ liposome encapsulated LSFV-IL-12 particles per m^2^ (Ren et al., 2003). The treatment caused no major toxicity. Patients receiving the higher dose had temporary and mild inflammatory reactions such as itching and slight fever with flu-like symptoms most likely due to enhanced IL-12 concentrations. In the peripheral blood 10-fold higher IL-12 concentrations were measured compared to the baseline, which lasted for 3–4 days.

In the case of ovarian cancer, SIN particles have demonstrated tumor targeting because of the overexpression of laminin receptors, which are utilized for host cell recognition by SIN (Tseng et al., 2004). The oncolytic SIN AR339 strain showed cytotoxicity and apoptosis in the HOC-1, HAC-2, and OMC-3 ovarian cancer cell lines (Unno et al., 2005). Moreover, in a metastatic ovarian cancer model ascites formation was suppressed after immunization of mice with SIN AR339. Alphaviruses have been subjected to combination therapy of ovarian cancers. For example, SIN-IL-12 particles administered together with the CPT-11 topoisomerase inhibitor irinotecan granted long-term-survival of SCID mice carrying highly aggressive human ES2 ovarian tumors (Granot and Meruelo, 2012). Furthermore, a prime-boost regimen with SFV expressing ovalbumin (OVA) and vaccinia virus (VV-OVA) was tested in C57BL/6 mice with implanted murine ovarian surface epithelial carcinoma (MOSEC) (Zhang et al., 2010). The immunization elicited OVA-specific CD8^+^ T-cell responses and improved the anti-tumor activity.

Pancreatic cancer therapy has also been of interest due to the poor prognosis of patients and lack of efficient treatments. Based on preclinical studies, where a single dose or a prime-boost regimen with VEE-CEA particles showed high levels of T-cell antibody responses, VEE-CEA particles were repeatedly administered intramuscularly into patients with metastatic pancreatic cancer in a phase I trial (Morse et al., 2010). The treatment induced clinically relevant T cell and antibody responses. The outcome was cellular cytotoxicity against tumor cells and prolonged overall survival in cancer patients. Irreversible electroporation (IRE), also called Nanoknife, has been combined with the oncolytic M1 alphavirus for the treatment of pancreatic cancer (Sun et al., 2021). IRE triggered apoptosis in pancreatic cancer cells (PCCs) and when combined with M1 administration, the therapeutic efficacy was synergistically enhanced, illustrated by inhibition of tumor proliferation and prolonged survival of immunocompetent mice with implanted orthotopic pancreatic tumors.

Alphaviruses have also been evaluated in the context of prostate cancer. VEE particles expressing the prostate-specific membrane antigen (PSMA) induced robust PSMA-specific immune responses in BALB/c and C57BL/6 mice (Durso et al., 2007). A single injection of 2 × 10^5^ VEE-PSMA particles elicited strong T- and B-cell responses, which were enhanced after repeated immunizations. Moreover, the immune responses were characterized by Th-1 cytokines, potent CTL activity and IgG2a/IgG2b antibodies. In another study, VEE particles expressing the mouse six-transmembrane epithelial antigen of the prostate (mSTEAP) were subjected to a booster immunization 15 days after the prime immunization with gold-coated conventional pcDNA-3-mSTEAP plasmids delivered by gene gun (Garcia-Hernandez et al., 2007). The immunization elicited specific CD8^+^ T-cell responses against a newly defined mSTEAP epitope and significantly prolonged the overall survival of mice subjected to tumor challenges. Moreover, the immunization strategy showed a modest but significant delay in growth of previously established tumors. In another approach, VEE particles expressing the prostate stem cell antigen (PSCA) were administered to transgenic adenocarcinoma of the prostate (TRAMP) mice (Garcia-Hernandez et al., 2008). The immunization demonstrated long-term survival in 90% of mice lasting for at least 12 months. VEE-PSMA particles have also been subjected to a phase I clinical trial in patients with castration resistant metastatic prostate cancer (CRPC) (Slovin et al., 2013). Immunization with 0.9 × 10^7^ or 3.6 × 10^7^ IU of VEE-PSMA particles showed good safety and tolerance. However, the PSMA-specific immune responses were disappointingly weak.

## Conclusion

In summary, alphavirus vectors have been evaluated for prophylactic and therapeutic use for a broad range of cancer indications in various animal models and in several clinical studies as summarized in Table 1. Generally, SFV, SIN or VEE vectors have been used although application of the oncolytic M1 alphavirus has also been described. The properties of SFV, SIN, and VEE vectors are very similar although for vaccine development it has been claimed that VEE shows targeting of dendritic cells *in vivo* providing superior immune responses (MacDonald and Johnston, 2000). However, it was determined that a single amino acid substitution in the E2 glycoprotein rendered dendritic cells susceptible to SIN infection (Gardner et al., 2000). Although based on numerous vaccine studies it has not been possible to demonstrate superiority of any alphavirus system regarding immune responses or therapeutic efficacy. In most cases robust immune responses have been obtained, including both humoral and cellular responses. The Th1-biased immunogenicity confirmed the potential of alphavirus-based cancer vaccine. The possibility to include alphavirus-based delivery of cytotoxic genes, anti-tumor genes, immunostimulatory genes, the apoptosis induced by alphaviruses, and RNA interference in the form of short interfering RNAs and micro-RNAs expands the possibilities of therapeutic interventions. Moreover, alphavirus vectors can be applied as recombinant viral particles, including replication-deficient, replication-proficient, and oncolytic viruses as well as RNA replicons and DNA replicons. It has been demonstrated that the stability of RNA and its resistance against degradation can be improved by RNA encapsulation in lipid nanoparticles. Several studies have also confirmed that due to the presence of alphavirus replicons, both RNA replicons and DNA replicons can induce the same immune response at 100–1000-fold lower doses compared to synthetic mRNA and conventional DNA plasmids, respectively. Although alphaviruses have demonstrated good safety and efficacy in various animal models, the transfer to humans have often generated disappointingly weak immune responses in clinical trials. A number of issues such as targeting, delivery, dose optimization and potential combination therapy still needs to be addressed.
